# Colpodellosis: Is *Colpodella* spp. an Emerging Tickborne Pathogen of Public Health Importance?

**DOI:** 10.3390/pathogens15060563

**Published:** 2026-05-23

**Authors:** Tobili Y. Sam-Yellowe

**Affiliations:** Department of Biological, Geological and Environmental Sciences, Cleveland State University, 2121 Euclid Avenue, SI 219, Cleveland, OH 44115, USA; t.sam-yellowe@csuohio.edu

**Keywords:** colpodellosis, tickborne diseases, apicomplexans, blood borne parasites, *Colpodella* species, emerging pathogens, myzocytosis, public health, relapsing fever, zoonoses

## Abstract

*Colpodella* spp. are phylogenetically related to apicomplexans such as *Plasmodium* spp., *Babesia* spp., and *Cryptosporidium* spp. *Colpodella* spp. are free-living protists that prey on bodonids, ciliates, and algae using myzocytosis. *Colpodella* spp. cause human and animal infections known as colpodellosis, with transmission via ticks across different geographic areas on different continents. *Colpodella* spp. DNA has been detected in ticks, the biting fly *Stomoxys indicus* and vertebrate samples using polymerase chain reaction (PCR). Ticks transmit zoonotic pathogens, and the identification of *Colpodella* spp. in animals poses a major public health risk due to human and animal encounters exposing humans to tick bites. However, it is unclear if ticks are confirmed vectors for *Colpodella* spp., since tick vector competence and capacity for *Colpodella* spp. transmission has not been experimentally demonstrated. Human cases of colpodellosis have involved three cases of blood infection, a fourth case of tickborne infection, and a fifth case of urinary tract infection. In this narrative review, the occurrence of *Colpodella* spp. in ticks that transmit zoonotic pathogens will be reviewed. Differences in the disease presentations and symptoms of colpodellosis in tickborne infections will be discussed. The pattern of *Colpodella* spp. coinfections with piroplasms and *Cryptosporidium* spp. will be evaluated. The pressing need for morphological identification of *Colpodella* spp. to assist proper characterization of the different species identified in arthropods and vertebrate hosts will be highlighted.

## 1. Introduction

*Colpodella* species are cosmopolitan free-living protist relatives of the pathogenic Apicomplexa. *Colpodella* spp. are typically characterized as predators that prey on other protists such as ciliates, bodonids, and algae by myzocytosis in the soil, freshwater, and marine environments [1,2,3]. *Colpodella* spp. have two life cycle stages: a fusiform biflagellated trophozoite with a curved rostrum at the anterior end and a cyst stage that undergoes cell division to release two or more juvenile trophozoites. *Colpodella* spp. share structural features with pathogenic Apicomplexa, such as rhoptries and micronemes, in life cycle stages including sporozoites and merozoites of *Plasmodium* and *Babesia* species [3,4]. Phylogenetic analyses place *Colpodella edax* and *Colpodella* sp. 50594 in a sister lineage to apicomplexans such as *Cryptosporidium serpentis*, *Babesia gibsoni*, *Theileria buffeli*, *Toxoplasma gondii*, *Caryospora bigenetica,* and *Eimeria alabamensis* [4]. Previous studies of the life cycle of *Colpodella* sp. ATCC 50594, a laboratory model, showed that intermittent stages occur during the life cycle in culture [5]. Juvenile trophozoites mature into older trophozoites, with both stages of trophozoites preying on *Parabodo caudatus*. During myzocytosis, trophozoites form a posterior food vacuole and develop into a pre-cyst stage [3,4]. Following degradation of the anterior end of the pre-cyst, encystation occurs, leading to the development of transient or permanent cysts [6]. Transient cysts excyst within 1–2 h in active culture, whereas permanent cysts persist for up to 14 days [5]. The life cycle in culture described for *Colpodella* sp. ATCC 50594 provides an important model for investigating life-cycle stage differentiation in environmental *Colpodella* spp. However, this life cycle may not represent the life cycle in the environment and within arthropod and vertebrate hosts. Further investigations are needed to understand the biology and life cycle of *Colpodella* spp. Following myzocytosis, most species of *Colpodella* encyst. *Colpodella unguis* and *C. pseudoedax* do not form cysts but divide by fission [7]. *Colpodella* sp. ATCC 50594 undergoes endocytosis as an alternative means of nutrient uptake, suggesting that in the absence of prey, *Colpodella* spp. can survive within arthropod and vertebrate hosts. However, it is unclear which macromolecules serve as nutrient sources and whether encystation occurs after endocytosis [8]. *Colpodella* spp. are reported to infect vertebrate hosts following tick bites, resulting in symptomatic and asymptomatic infections and posing a public health risk to humans that encounter infected animals [9,10,11,12,13,14]. The magnitude of the public health threat of *Colpodella* spp. infection is unknown and needs to be investigated to determine transmission, virulence, and pathogenic mechanisms. Tick vector competence needs to be investigated to confirm ticks as either biological or mechanical vectors for *Colpodella* spp. transmission. Although there is much that is unknown, reported cases of human and animal infections where *Colpodella* spp. was the only organism identified following PCR amplification of DNA from host samples suggest *Colpodella* spp. may be an agent of infection [10,11,12,13,14]. However, further studies are needed to verify infectivity of *Colpodella* spp.

**Figure 1:**
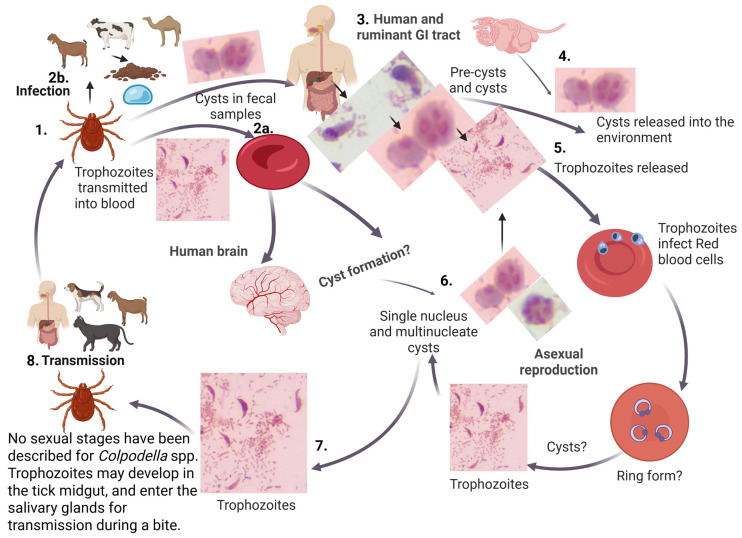
Proposed hypothetical life cycle and transmission of tick-borne colpodellosis shown using *Colpodella* sp. ATCC 50594 life cycle stages, formalin fixed and stained with Sam-Yellowe’s trichrome A stain. (1) Tick-borne transmission can occur through tick bites with the deposition of *Colpodella* spp. trophozoites into the bloodstream of the vertebrate host. (2a) Trophozoites can invade red blood cells for intracellular infection or remain in the blood extracellularly for transfer into tissues like the brain. (2b) Ticks may pick up *Colpodella* spp. trophozoites or cysts from water containing *Colpodella* spp., or direct transmission can also occur through contaminated water containing *Colpodella* spp. trophozoite and cysts deposited into the environment through fecal samples from infected hosts such as ruminants, felines, cows, or camels. Trophozoites may encyst in tissues and after cell division, release two or more trophozoites. Released trophozoites may encyst in the GI tract (3). Excystation leads to trophozoite release, which can differentiate after feeding into pre-cysts, then into cysts, which release trophozoites after excystation and are then released into the environment (4). Trophozoites may reinvade red blood cells or remain extracellular in the tissues and in the blood. The trophozoites may invade red blood cells to form intracellular ring-like stages (5) or differentiated trophozoites may encyst (6). Trophozoites released through excystation can continue infection in the blood, or ticks can pick up circulating trophozoites during a tick bite (7) for transmission to a new host (8). Created in https://BioRender.com (accessed 4 April 2026).

A proposed life cycle for *Colpodella* spp. transmission is shown in Figure 1. Whether tick-associated infections or direct infections occur, as shown, will need to be investigated and confirmed. No studies have been performed to verify vector competence for *Colpodella* spp. transmission. Since sexual stages are unknown in the life cycle of *Colpodella* spp., comparisons to the life cycles of *Babesia* spp. and *Thelieria* spp. remain cautious. The proposed life cycle shown in Figure 1 is speculative and intended to guide investigations examining specimens obtained from vertebrate animals and arthropods in identifying *Colpodella* spp. life cycle stages mediating transmission and are responsible for pathogenesis. The ability to perform collaborative investigations is crucial to enable labs that are not set up for microscopy to collaborate with labs that perform microscopy on a routine basis, to facilitate morphological identification of *Colpodella* species. Based on the number of cases currently reported in humans and animals, and the risk to public health due to transmission of infection by ticks, ignoring the morphological characterization of *Colpodella* spp. identified in epidemiological screening and survey studies slows the progress of understanding the infection dynamics of colpodellosis and the etiological agent for the disease.

*Colpodella* species DNA has been detected in symptomatic and asymptomatic vertebrate hosts and has also been reported in six genera of ticks and in a biting fly [10,11,12,13,14]. The presence of *Colpodella* species in blood, cerebrospinal fluid (CSF), urine, and fecal samples of infected hosts detected by DNA amplification using PCR suggests different routes of transmission and possibly host specificity [10,11,12,13,14,15].

The combination of morphological identification of *Colpodella* spp. and strains by microscopy, immunoassays, molecular markers, and virulence markers will be needed to confirm and distinguish active infection, transient presence, and environmental contamination of collected specimens. However, the major source of transmission, with accompanying signs and symptoms, has been ticks. Transmission through contaminated drinking water, followed by gastrointestinal illness resulting in diarrhea, has also been proposed, albeit with a link to ticks acquiring *Colpodella* spp. from contaminated water [11,15]. Although uptake of water contaminated with *Colpodella* spp. by ticks appears an unlikely source for transmission, ticks have been described to directly drink water using their mouthparts as well as take up water through water vapor uptake and absorption [16]. Secretion of a hygroscopic solution containing chlorine, sodium, and potassium from the tick salivary glands aids in the absorption of water vapor [16], allowing for the maintenance of water balance in the ticks. In direct water-uptake studies, fluorescent dyes and bacteria in the water droplets were taken up by ticks, with the bacteria identified in tissues of the ticks [16]. Although this data suggests that protists like *Colpodella* spp. may be taken up through water, it is crucial that the transmission routes and mechanisms of *Colpodella* spp. infection be investigated to identify routes and mechanisms of transmission.

*Colpodella* spp. and strains identified in ticks share identity with species and strains identified in humans and other vertebrate hosts, indicating that colpodellosis is a zoonotic infection. The number of cases of tick-borne disease in humans and animals continues to increase. From 2012 to 2020, six studies reported *Colpodella* spp. DNA detection by PCR in ticks and animals [reviewed in [9]. Two of the reports identified *Colpodella* spp. DNA in human blood and CSF through DNA amplification by PCR using 18S rDNA primers. From 2021 to 2026, twenty cases of *Colpodella* spp. detection in infected animals, and three human cases were reported [9]. In 2025 alone, seven cases of *Colpodella* spp. detected by PCR amplification of DNA were reported. Four reports from China, one from Cyprus, and one from Egypt [9]. *Colpodella* spp. detected by DNA amplification in arthropods and vertebrates has been reported from countries in Africa, Asia, and Europe [9]. The type of animals in which *Colpodella* spp. DNA has been detected as expanded to include the Chinese mole shrew (*Anourosorex squamipes*, Accession number PX220039), Dracula shrew (*Crocidura dracula*, Accession number PX220011), Yunnan red-backed vole (*Eothenomys miletus*, Accession number PX220013), and the domestic water buffalo (*Bubalus bubalis*, Accession number PX220025), a host for *Theileria* spp. and *Babesia* spp. While *Colpodella* spp. PCR-based DNA detection has aided diagnosis; no information is available on the morphological characteristics of *Colpodella* spp. identified in ticks, blood, CSF, and fecal samples.

In this narrative review, the occurrence of *Colpodella* spp. in ticks that transmit zoonotic pathogens will be reviewed. Differences in disease presentations and symptoms of colpodellosis among infected vertebrate hosts will be discussed. Factors influencing tick vector competence will be discussed, and the pattern of *Colpodella* spp. coinfections with piroplasms and *Cryptosporidium* spp. will be evaluated. The pressing need for morphological identification of *Colpodella* spp. to assist proper characterization of the different species identified in arthropods and vertebrate hosts will be highlighted. Morphological identification of *Colpodella* spp. identified in ticks and vertebrate hosts, along with nucleic acid amplification, immunoassays, and the use of molecular markers, is of vital importance in understanding the biology of tickborne colpodellosis.

## 2. Human and Animal Colpodellosis

*Colpodella* spp. has been identified in co-infections with *Babesia* spp., *Theileria* spp. [17,18] and *Cryptosporidium* spp. [19,20] using primers targeting the 18S rRNA gene of these pathogenic apicomplexans. There is an urgent need to identify the morphology of *Colpodella* spp. detected in human and animal hosts, to ensure accurate identity of infecting species, since there is currently a lack of specific molecular probes to identify *Colpodella* spp. The use of PCR primers targeting the 18S rRNA gene can result in amplification of DNA from non-target organisms. Cross-amplifications can also occur, and without morphological confirmation of the target organisms, may result in misleading data. Specific DNA sequences and other specific biomarkers are unavailable for the detection of *Colpodella* spp., further intensifying the urgency for studies on the cell biology of *Colpodella* spp. Currently, studies reporting *Colpodella* spp. DNA detection in a single tick infesting a vertebrate host draws attention to the occurrence of potential host infections. The small sample size in these situations raises questions about the vector capacity of the tick host. The use of DNA amplification by PCR and next-generation sequencing (NGS), although sensitive in their application, can detect contaminant or environmental DNA, further emphasizing the need to use integrated methods to detect and study the biology of *Colpodella* spp. Human and animal hosts infected with *Colpodella* spp. present varied symptoms. Understanding the types of symptoms and associating them with specific sites of infection in tissues of infected hosts will aid accurate diagnosis. In human infections, fever, anemia, headache, and neurological symptoms have been described [14,21,22]. The prospects for zoonotic infections with serious impact on public health continue to increase as new cases are reported in animals that come into close contact with humans in recreational, domestic, and farm environments, and for humans encountering infected wildlife and animals housed in zoos [12,19,20,23,24,25,26].

Among the pathogenic apicomplexa, intracellular infections occur in blood cells, such as erythrocyte infection by *Plasmodium* spp. and *Babesia* spp., and *Theileria* spp. infection in lymphocytes. *Toxoplasma gondii* infects macrophages and other nucleated cells, and *Cryptosporidium* spp. infect epithelial cells in the gastrointestinal tract [27]. The life cycles of the apicomplexa include asexual and sexual stages. Whether transmission is through a vector such as mosquitoes or ticks for *Plasmodium* and *Babesia* transmission, respectively, the life cycle stage initiating infection is the sporozoite stage. Similarly, ingestion of oocysts of *Cryptosporidium* spp. and coccidians results in the release of sporozoites that initiate the infection. Sporozoite invasion is followed by schizogony (merogony) and the release of merozoites, which can maintain the asexual stage infection or differentiate into male and female gametocytes, which, after fertilization, develop into a zygote that matures into the oocyst [27]. The gametes of both *Theileria* spp. and *Babesia* spp. formed in the vertebrate host, fuse to form zygotes in the tick midgut, followed by differentiation into motile elongated kinetes. The kinetes enter the tick hemolymph and invade tissues like the ovaries [27]. Currently, there is no indication that the life cycle and transmission of *Colpodella* spp. is the same or similar to that of the piroplasms. Still, it does provide a starting point for which serious questions can be asked and investigated to understand the biology of *Colpodella* spp., vector carriage, and the mechanism of host infection. Despite the reports of human and animal transmission of *Colpodella* spp., the life cycle stage initiating infection or causing transmission is unknown. Similarly, life cycle stages responsible for pathogenesis in infected hosts are unknown. It is unclear how *Colpodella* spp. survives in arthropods and in vertebrate hosts. *Colpodella* spp. are predatory protists in the environment, feeding on ciliates, bodonids, and algae.

Currently, it is unknown whether *Colpodella* spp. is present with its prey in both arthropod and vertebrate hosts, or whether *Colpodella* spp. invades host cells or performs endocytosis to obtain nutrients. Although the life cycle of the model *Colpodella* sp. ATCC 50594 has been described in culture [5]; the life cycle in the environment is unknown. Whether infected humans and animals are dead-end hosts is unknown. However, the presence of *Colpodella* spp. in blood and in fecal samples suggests that life cycle stages are introduced back into the environment from infected hosts through ticks and biting flies and through the presence of life cycle stages in fecal samples [11,12,13,15,20]. Transmission may be maintained by tick and fly bites and by drinking contaminated water or possibly ingesting cyst-contaminated food, like transmission of other cyst-forming pathogenic protozoa [27].

Seventy-five percent of infections caused by viral, bacterial, and parasitic pathogens are zoonotic diseases [28]. Tick-borne infections contribute to zoonotic diseases and pose a major public health risk due to close associations between infected animals and humans. Human-to-human, animal-to-human, and human-to-animal infections can be maintained through tick bites with either biological development of the pathogen within the ticks or mechanical transfer of the pathogen by the tick. If the development of *Colpodella* spp. within the vertebrate hosts follows what is known for other cyst-producing pathogenic protozoans, we would expect the development of cyst stages within the infected host [27]. Cyst stages of protozoans like *Entamoeba*, *Giardia,* and oocysts of *Cryptosporidium* spp. develop in the gastrointestinal (GI) tract and are eliminated into the environment with feces [27]. Alternatively, if active trophozoite stages are introduced into the host through tick or fly bites, these same stages can also be picked up by the arthropods for transmission to new hosts. Currently, it is unclear whether infections in vertebrate hosts are intracellular or extracellular. The first report of human infection by *Colpodella* spp. was described as an intracellular infection due to the detection of *Colpodella* within erythrocytes by Giemsa staining [21]. Confirmation of intracellular infection by *Colpodella* spp. will require electron microscopy data. *Colpodella gonderii* and its prey *Colpoda steinii* were detected in human urine by Giemsa stain [29] in a urinary tract infection. In additional reports of human and animal infections, *Colpodella* spp. DNA was detected in blood, CSF, fecal samples, and in ticks by PCR, DNA sequencing, and phylogenetic analysis after sequence alignment [9]. There have been no reports on the morphology of *Colpodella* spp. from infected hosts following tick or fly bites and from fecal samples. The identification of *Colpodella* spp. in human and animal infections was initially considered a rare, opportunistic, or “accidental” occurrence [9,25]. However, the number of infections where *Colpodella* spp. are the only organism identified by PCR, where ticks are involved in transmission, and where symptoms were described has increased [11,14]. Phylogenetic analysis of identified DNA sequences suggests different infective species and strains. While the term “opportunistic” infections may be applied, colpodellosis is a tick-borne infection, with some *Colpodella* species causing relapsing fever and GI tract infections. Further investigation is needed to understand the transmission of *Colpodella* spp. and the dynamics of infection.

## 3. Microscopy and Morphological Characterization of *Colpodella* Species

Diagnosis and management of parasitic infections require an integrated effort combining microscopy, nucleic acid amplification methods like PCR and DNA sequencing, CRISPR/Cas 12a, serology, immunoassays, and cell culture [8]. This is of importance in the screening and diagnosis of emerging pathogens. Microscopy is the gold standard for the diagnosis of parasitic and infectious disease-causing organisms. Identification of intracellular and extracellular parasites in blood and other body fluids relies on wet mounts and stained smears examined by light microscopy. Microscopy remains a fundamental and the most valuable tool for the identification of parasites [30,31,32,33]. Blood or CSF smears containing trophozoite stages of parasites can be observed after staining with Giemsa. The presence of life cycle stages in tissues can also be identified using hematoxylin and eosin (H&E) staining. Light microscopy provides an easily available and accessible resource to accurately identify parasites. Microscopy is indispensable in identifying trophozoites and microfilariae in blood smears and in identifying cysts, oocysts, eggs, and helminth larvae in fecal samples. The combination of microscopy and molecular techniques remains critical for identifying new parasite species [34,35]. For colpodellosis, identifying the morphological characteristics of *Colpodella* spp. detected in ticks and in infected hosts is vital in the absence of specific molecular probes for *Colpodella* spp.

**Figure 2:**
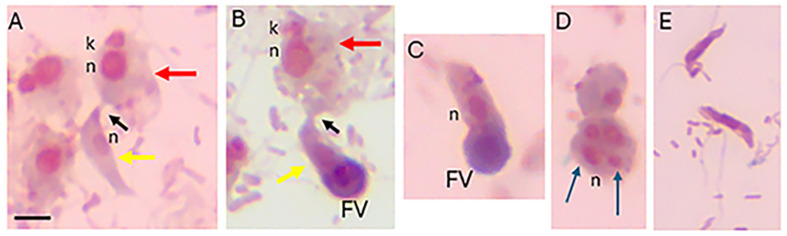
Sam-Yellowe’s trichrome A staining of *Colpodella* sp. ATCC 50594 life cycle stages in nutrient uptake cultures. *Colpodella* sp. ATCC 50594 trophozoite attached to *P. caudatus* feeding by myzocytosis, shown in (**A**,**B**). Yellow arrows identify *Colpodella* sp., the red arrow identifies the prey *P. caudatus,* and the black arrow identifies the tubular tether formed between predator and prey during myzocytosis. A large posterior food vacuole (FV) forms during the feeding process (**B**), resulting in the formation of a pre-cyst at the conclusion of myzocytosis (**C**). Encystation occurs followed by cell division to form a single-nucleus cyst or cysts containing four or more nuclei. The blue arrows identify a four nuclei cyst (**D**). Juvenile trophozoites are released following cell division (**E**). The images shown were captured at ×1000 magnification. Nucleus (n), kinetoplast (k), and food vacuole (FV). The images are unpublished from the Sam-Yellowe lab archives. Panel D is used in Figure 2 to show a single-nucleus and a four-nuclei stage cyst. (Scale bar: 10 µm).

The continued detection of *Colpodella* spp. DNA using PCR primers targeting the piroplasms *Babesia* spp., *Theileria* spp., and *Cryptosporidium* spp. requires the development of specific molecular and morphological protocols for *Colpodella* spp. identification. Misidentifications and misdiagnoses can occur without integration of diagnostic techniques. There is a long history of parasite research, particularly among protozoan parasite research, which provides an opportunity to understand *Colpodella* spp. stages transmitted to vertebrate hosts through ticks and biting flies [10,11,12,13,14], and to understand the vector capacity, competence, and biological necessity of arthropods as vectors for colpodellosis. The advantages of microscopy outweigh the disadvantages, particularly the rapid identification of parasites in wet mounts or stained preparations and the low cost of light microscopy. Often cited disadvantages include the need for skilled microscopists, challenges in identifying parasites when parasitemia or parasite density is low, as may be the case in environmental samples, and the need to determine appropriate staining techniques [30,31,32,33]. To overcome the challenges of identifying cysts of *Colpodella* sp. ATCC 50594 and its prey, *P. caudatus,* using Giemsa staining, Sam-Yellowe’s trichrome staining series was developed to aid *Colpodella* spp. life cycle stage identification [9]. The staining protocol allowed identification and differentiation of *Colpodella* sp. ATCC 50594 and *P. caudatus* cysts in culture, and allowed identification of life cycle stage transitions during the life cycle of *Colpodella* sp. ATCC 50594 in culture [36] (Figure 2). Sam-Yellowe’s trichrome staining was also used to stain *Cryptosporidum* spp. oocysts from commercial slide preparations, *Plasmodium falciparum* from in vitro cultures, and *Voromonas pontica* in culture to demonstrate the utility of the staining protocol in identifying parasite life cycle stages in low-density samples [36]. Diagnosis, differentiation of life cycle stages in ticks and infected hosts, and quantitation of parasite density require microscopy. Furthermore, investigating interactions of *Colpodella* spp. with host cells and accurate taxonomic investigations require microscopy.

There are numerous unanswered questions regarding the mode of parasite transmission in colpodellosis. The life cycle stages initiating transmission, causing pathogenesis, and maintaining spread within and among hosts are unknown. Based on the literature reports of colpodellosis, the routes of transmission shown in Figure 1 are proposed to enhance needed investigations. The hypothetical steps for transmission shown in Figure 1 suggest similarity to the transmission pattern known for piroplasms. The actual pattern and mechanism of transmission for *Colpodella* spp. may differ from the proposed sequence shown in Figure 1. Morphological studies using light and electron microscopy with the lab model *Colpodella* sp. ATCC 50594 has been identified with developmental stages in the life cycle in culture, as shown in Figure 2. It is unclear if similar developmental stages are found in the different species of *Colpodella*. In the proposed pathway, tick-borne transmission may occur via tick bites, with deposition of *Colpodella* spp. trophozoites into the bloodstream of the vertebrate host, leading to intracellular erythrocyte infection, as reported in the human case of relapsing fever [21], or trophozoites may remain in the blood extracellularly for transfer into tissues such as the brain [14]. Differentiation of the trophozoites into the cyst stage may occur in tissues, and after cell division, trophozoites are released, reinvade red blood cells, or remain extracellular in the tissues and in blood, where a feeding tick can pick up circulating trophozoites for transmission to a new host. As proposed in Figure 1, ticks may also pick up *Colpodella* spp. from water [11,15]. 

Uptake of *Colpodella* spp. from contaminated water by ticks may be an unlikely route for transmission due to the mode of water uptake used by ticks through the uptake of water vapor [16], which may not contain pathogens. Direct transmission may also occur through drinking contaminated water containing *Colpodella* spp. trophozoite and cysts deposited into the environment through fecal samples from infected hosts such as ruminants, felines, cows, or camels [11,20,25,26]. Within the GI tract, excystation leads to the release of trophozoites, which differentiate into pre-cysts, then cysts, which can be released into the environment. Excystation may occur after cell division, and trophozoites released can enter the bloodstream, infect red blood cells for intracellular infection, or remain extracellularly in the blood. Trophozoites differentiate into cysts, which undergo cell division to release trophozoites that will maintain the infection. Trophozoites in the blood can be picked up by ticks, which can transmit *Colpodella* spp. to a new host.

Two key questions remain: how does *Colpodella* spp. survive within the arthropod and vertebrate hosts? Are the ciliate or bodonid prey also present in the arthropod and vertebrate hosts? *Babesia bigemina* is transmitted by *Rhipicephalus* (*Boophilus*) *annulatus*, a one-host tick that transmits the parasite to developing eggs in the ovaries in a process known as transovarial transmission. Feeding, maturation, and mating of the tick occur on one host. After the female tick feeds, mates, and drops to the ground, she lays eggs and dies. Larvae develop from eggs, climb to the top of vegetation, and attach to hosts that brush against them [27]. *Theileria* spp. can be transmitted by one-, two-, and three-host ticks. Transmission of *Theileria* spp. by *Rhipicephalus appendiculatus*, a three-host tick, results in all instars of the tick (larvae, nymph, adult) feeding on different hosts [27]. Whether *Colpodella* spp. life cycle stages follow the same developmental pattern as *Babesia* spp. or *Theileria* spp. within the tick is unknown and needs to be investigated. *Colpoda steinii* was identified with *Colpodella* spp. in two infections [26,29]. 

In addition, *P. caudatus* and *Bodo* spp. were identified with *Colpodella* spp. in a blood infection [37]. It is also unknown whether *Colpodella* spp. trophozoites can feed on host cells directly or endocytose nutrients from host fluids. Protozoan parasites invading host tissue have been described to feed by trogocytosis, phagocytosis, and phagotrophy, resulting in tissue damage and pathogenesis [38,39]. Mechanisms of virulence and pathogenesis are unknown for *Colpodella* spp. Whether *Colpodella* spp. carry viruses and bacteria that may contribute to pathogenesis, like pathogenic amoeba, is unknown [40,41]. The mechanisms by which *Colpodella* spp. are maintained and transferred among the developmental stages of the tick are unknown. The proposed hypothetical life cycle shown in Figure 1, with the proposed routes of transmission, is speculative since there are gaps in the knowledge of *Colpodella* spp. biology, are aimed at stimulating further investigations among investigators performing epidemiological screenings for tick-borne pathogens. The route of entry and spread of *Colpodella* spp. life cycle stages need to be identified. The sites of infection within tissues and the stages responsible for pathogenesis need to be identified and life cycle stage differentiations with the tick and vertebrate hosts need to be identified. Microscopic evaluations of collected samples, staining for morphological characterizations of *Colpodella* spp. life cycle stages present in collected samples will aid investigations aimed at determining if the blood infections currently described as non-tick-associated involve tick bites. Culturing and staining protocols along with integrated diagnostic platforms for morphological and molecular characterization for *Colpodella* spp. were reviewed previously [9]. Furthermore, the identification of the RhopH3, Kelch 13, and coronin genes in *Colpodella* sp. 50594 by PCR and immunofluorescence [9] demonstrates that additional genes whose products are involved in myzocytosis, nutrient uptake, and virulence can be identified. Efforts should be made to identify virulence markers for *Colpodella* spp. in tickborne, blood, and GI infections. Whole genome sequencing of *Colpodella* species will identify specific markers that will clarify *Colpodella* species identity in specimens. Specific probes and primers targeting *Colpodella* nucleic acids in amplification protocols can be constructed. This will provide important clarifications on why colpodellosis is symptomatic in some hosts but not in others.

## 4. Symptomatic and Asymptomatic Tickborne Colpodellosis

### 4.1. Symptomatic Infections

Mosquitoes and ticks are the most important arthropod vectors that transmit pathogens, including viruses, bacteria, protozoa, and helminth parasites. Mosquitoes transmit most vector borne pathogens, with ticks representing the next most frequent arthropod vector [42]. *Colpodella* spp. have been reported in six genera of ticks known to transmit the piroplasmids *Babesia* spp. and *Theileria* spp. [9] (Table 1). Four species of *Hyalomma,* including the newly identified vectors *Hy. anatolicum* and *Hy. Excavatum*, carry *Colpodella* spp. [43]. *Rhipicephalus annulatus* was identified in Egypt for the first time, carrying *Colpodella* spp. in cattle infestation [44]. Altogether, five species of *Rhipicephalus* carry *Colpodella* spp., and five species of *Dermacentor* carry *Colpodella* spp. and transmit zoonotic pathogens of public health importance [9] (Table 1). Host symptoms vary in humans and animals reported to be infected with *Colpodella* spp. following detection of *Colpodella* spp. DNA by PCR in blood, CSF, and fecal samples. In some symptomatic infections, *Colpodella* spp. was the only organism identified (Table 2). 

In a tick-borne human infection, a 55-year-old female Chinese patient from Heilongjiang Province of Northeast China presented with neurological symptoms following a tick bite [14]. Fever, dizziness, headache, and gait disturbance were among the symptoms reported. Although suspected to have Lyme disease, no ulceration, exudate, or erythematous lesions were observed [14]. Blood and CSF were examined by PCR using primers targeting the 18S rRNA gene of *Babesia* spp. Other suspected pathogens in the patient were *Eperythrozoon* spp., *Borrelia* spp., *Anaplasma* spp., *Ehrlichia* spp., *Rickettsia* spp., and tickborne encephalitis virus (TBEV). *Colpodella* spp. was the only pathogen identified in the patient’s CSF by PCR amplification of *Colpodella* spp. DNA. However, no *Colpodella* spp. DNA was detected in the patient’s blood. This raises important questions about the dynamics of infection, tissue tropism of *Colpodella* spp. life cycle stages in hosts and the molecular markers aiding the spread of *Colpodella* in host tissues. Studies are required to understand the infection dynamics of *Colpodella* spp. in ticks and vertebrate hosts. *Colpodella* spp. was identified in 2/474 adult *Ixodes persulcatus* ticks collected in woodlands around the patient’s home. Treatment with doxycycline (used for malaria prophylaxis and treatment) resolved the infection. Jiang et al. [14] reported the detection of anti-*Borrelia burgdorferi* antibodies. However, PCR was negative for *B. burgdorferi* DNA. The *Colpodella* spp. strain HLJ identified from CSF shared 88.0–89.0% identity with the tick *Colpodella* spp., and both tick *Colpodella* DNA (accession numbers KT600661 and KT60062) shared 93.8% identity with each other.

An infection was reported in a tick-infested South China tiger (*Panthera tigris amoyensis*) from the Meihua Mountains, China [11] bitten by *Haemaphysalis flava*. The only pathogen identified was *Colpodella* spp. PCR for the detection of *Mycoplasma suis* and *T. gondii* DNA was negative. The tiger showed symptoms of anorexia, a runny nose, drool, and had bluish-green stools and was treated with sulfonamide, cephalosporin, and ampicillin [11]. DNA extracted from ticks collected from the tiger, and from tiger blood, was amplified using nested PCR with universal primers targeting the 18S rRNA gene of piroplasmids. Following the death of the tiger, hepatomegaly, splenomegaly, hemorrhage in the kidneys and mesenteric lymph nodes were observed, and pathology revealed severe, whole-body jaundice of the skin, eyes, conjunctiva, oral mucosa, trachea, and coronary fats around the heart [11]. Three genera of ticks identified were found to be positive for *Colpodella* spp. Twenty-two *Colpodella* DNA sequences were identified among the three tick genera. Following DNA sequence alignment, the tiger and tick sequences had 100% identity. The sequences shared 90.1% sequence identity to *Colpodella* spp. HEP and 90.4% sequence identity to *Colpodella* spp. HLJ.

Among the ticks collected, 16/22 ticks carrying *Colpodella* spp. were *Haemaphysalis* spp. The dominant species were *H. flava* and *H. longicornis*. *Colpodella* spp. was also detected in water from ditches around the tiger enclosure. However, *Colpodella* spp. was not detected in the soil. Chiu et al. [11] suggested that ticks acquired *Colpodella* spp. from contaminated water and then transmitted the parasites to vertebrate hosts. A similar suggestion was made about *Colpodella* spp. detected in a red fox, regarding transmission of *Colpodella* spp. to the foxes by contaminated drinking water [15].

*Colpodella* spp. DNA was detected by PCR in ticks infesting two-humped camels (*Camelus bactrianus*) and in blood collected from symptomatic animals, in Gaotai County, Gansu Province, China [25]. Two hundred and eighty-eight ticks consisting of *Hyalomma asiaticum* (245/288) and *Haemaphysalis longicornis* (34/288) were collected from camels. One hundred and fifty blood samples were also collected from camels. *Hyalomma asiaticum* was the most predominant tick at 245/288 ticks examined. *Colpodella* spp. coinfections with bacteria were detected in ticks. Coinfections of *Colpodella* spp. and *A. bovis* (14/288), *Colpodella* spp. and *Rickettsia* spp. (1/288) and *Colpodella* coinfections with *Rickettsia* and *A. bovis* (1/288) were also detected. Infected camels exhibited symptoms of fever, appetite loss, diarrhea, fatigue, and decreased milk output. Although morphological identification and biochemical characterizations were carried out for the ticks, no morphological characterization of *Colpodella* spp. was performed.

Coinfections of *Colpodella* spp. and the bacteria *Rickettsia* and *Ehrlichia* were detected in *Ambyloma javanense* ticks infesting 17/21 rescued sick Malayan pangolins [9]. The pangolins were examined for ectoparasites and DNA from ticks, pangolin blood, and tissues of the sick pangolins following their death were amplified for virus, bacteria, and protozoan parasite DNA. The pangolins exhibited symptoms of anorexia, cough, edema of the extremities, and drowsiness. The animals also had bloody stools, hematuria, convulsions, and other neurological symptoms [10]. At autopsy, severe organ damage was observed, congestion, edema of major organs, ascites, and inflammation were observed. Histological examination of the pangolin tissues by H & E staining did not detect pathogens. DNA from *Colpodella* spp., *Rickettsia* spp., *Anaplasma* spp., *Ehrlichia* spp., and *Babesia* spp. was detected in ticks by PCR. *Colpodella* spp. was not detected in pangolin tissues. *Theileria* spp., *Hepatozoon* spp., and viruses were also not detected. Coinfections of *Colpodella* spp. and *Rickettsia*, and *Colpodella* spp., along with *Rickettsia* spp. and *Ehrlichia* spp., were detected [10]. Six out of 33 ticks carried *Colpodella* spp., which shared identity with 18S rRNA gene sequences from *Colpodella* spp. identified from Qinghai (MH012046) and Yunnan (MH208621), and from *Colpodella* HLJ strain identified from a human infection with neurological symptoms [14].

### 4.2. Asymptomatic Infections

Asymptomatic hosts infected with *Colpopdella* spp. pose an important public health risk since the parasite is present in the infected host, but due to the lack of symptoms, precautionary or preventive measures might not be taken to prevent parasite transmission to human hosts encountering infected animals or arthropod vectors. Asymptomatic animals serve as reservoirs that can maintain infections within communities and in the environment. Epidemiological studies involving screening of domestic, agricultural, and wildlife animals for tickborne pathogens and *Cryptosporidium* spp. have revealed the presence of *Colpodella* spp. in asymptomatic and symptomatic infections (Table 2). Jimale et al. [45] screened 98 cattle and 104 goats for tick-borne parasites. These were free-grazing animals from the Puglia region, Southern Italy. Genomic DNA was extracted from animal blood and from the ticks infesting them. Template DNA was amplified using primers targeting 18S rRNA from *Babesia* spp. and *Theileria* spp. Primers targeting the *gltA* gene of *Rickettsia* spp. were also used for PCR. *Babesia* spp., *Theileria* spp., and *Rickettsia* were detected in ticks. The predominant (31/42) tick identified among 42 adult male and female ticks collected from cattle was *Rhipicephalus bursa*. *Rhipicephalus secundus* was also identified (11/42). *Colpodella* spp. DNA with 100% identity to *Colpodella* spp. accession number OQ540588.1 was identified in one female *Rh. bursa*. Among the goats, the predominant tick species identified was *Rh. bursa* (25/36) along with *Rh. secundus* (11/36). *Rickettsia* spp. was identified in one female *Rh. bursa. Colpodella* spp. DNA was detected in asymptomatic hosts screened for tick-borne and blood-borne pathogens using PCR, suggesting that these hosts can serve as reservoirs to maintain zoonotic transmission between animal and human hosts. Blood was collected from asymptomatic pet dogs and cats attending a veterinary hospital in Guiyang, China [26]. Genomic DNA extracted from the collected blood was screened for piroplasmids using primers targeting the 18S rRNA gene. In pet cats, *Theileria uilenbergi*, *T. luwenshuni,* and *Colpodella* spp. were detected. In pet dogs, *T. uilenbergi* and *Colpodella* spp. were detected. *Colpodella* spp. was also detected in the tick *H. longicornis*. Although *Theileria* spp. do not cause human infections, the presence of *Colpodella* spp. raises public health concerns for zoonotic transmission to members of households with infected pets.

Qi et al. [17] performed epidemiological screening of asymptomatic dogs and goats for tickborne parasites in Yiyuan County, Central Shandong Province, China. Following PCR amplification of DNA extracted from *H. longicornis* ticks using primers targeting the 18S rRNA gene of *Theileria* spp. and *Babesia* spp., *Colpodella* spp. was identified in individual infections and from the ticks collected from goats and dogs. The *Colpodella* spp. DNA had 92–98% sequence identity to *Colpodella tetrahymenae* (accession number MH208619.1).

Two hundred asymptomatic camels from Southern Egypt were investigated for tick infestation, leading to the identification of *Hyalomma dromedarii* ticks on the camels [12]. The examination was performed during a routine veterinary evaluation of the camels. Two hundred and ninety-seven ticks identified on the camels were screened for tick-borne parasites. In 30/297 ticks, *Colpodella* spp. was identified using primers targeting the 18S rRNA gene of piroplasmids, and in 16/297 ticks, *Babesia bovis* was detected using primers targeting the spherical body protein-4 gene. *Colpodella* spp. was identified in *Rhipicephalus annulatus* infesting cattle in Egypt for the first time [44]. Two hundred and fifty-eight ticks were collected from 110 cattle during routine veterinary examinations. Genomic DNA was extracted from pooled ticks and screened for piroplasmids and *Colpodella* spp. using PCR. The major merozoite surface antigen gene of *Theileria annulata* identified *Theileria*, the rhoptry-associated protein 1 gene identified *Babesia bigemina*, the spherical body protein gene identified *B. bovis,* and the 18S rRNA piroplasm gene identified *Colpodella* spp. Coinfections of *Colpodella* spp. and *B. bovis*, *Colpodella* spp. and *T. annulatus,* and *Colpodella* spp., *B. bovis,* and *T. annulatus* were identified in the ticks. The minimum infection rate (MIR) of *Colpodella* detected in *Rh. annulatus* was 2.3% per sample of the pooled ticks examined [42].

*Colpodella* spp. was detected in the ticks *Hyalomma excavatum* and *Hy. anatolicum* infesting goats (*Capra hircus*) in Pakistan [43]. Ticks collected from goats from seven districts of Khyber, Pakhtunkhwa, Pakistan, were screened for pathogens. Among the pathogens, *Colpodella* spp., *Ehrlichia* spp., *Rickettsia hoogstraalii,* and *Providencia rettgeri* were identified with *Colpodella* spp. having a high prevalence in *Hy. excavatum* collected from Buner (15/167 ticks; 8.98%) and Kohistan (9/164 ticks; 5.48%) and in *Hy. anatolicum* from Chitral (8/100 ticks; 8%) [43]. *Colpodella* spp. from *Hy. anatolicum* had 100% sequence identity to *Colpodella* spp. (MH208621) from *Rhipicephalus haemaphysaloides* in China and 99.92% identity with *Colpodella* spp. (GQ411073.1) isolated from a woman with relapsing fever [21] and 99.59% identity to *Colpodella* spp. (accession number MH012046.1) isolated from *Dermacentor nuttalli* in China. In phylogenetic analysis, *Colpodella* spp. detected by Ullah et al. [43] clustered with *Colpodella* spp. identified from *Rh. annulatus* in Egypt (accession number PP937594), *Colpodella* spp. (accession number MH208620) in China, Luxembourg, Canada, and an uncultured alveolate from Kenya and Austria, and an uncultured eukaryote from France (accession number AY817009).

## 5. Symptomatic and Asymptomatic Blood Infections

### Symptomatic Infections

A case of relapsing fever was reported by Yuan et al. [21] in a 57-year-old female patient with a natural killer cell deficiency, in Yunnan Province, China [14]. She presented with a babesiosis-like blood infection and exhibited symptoms of malaise, productive cough, hemolytic anemia, and relapsing illness. Elevated reticulocytes and lactate dehydrogenase were also reported. PCR primers targeting conserved DNA fragments of *Babesia* 18S rRNA gene amplified DNA whose sequence had homology to *Colpodella tetrahymenae*. Giemsa staining and immunofluorescence assay identified intracellular infection in erythrocytes, and anti-*Colpodella* antibody reacted with *Colpodella* in erythrocytes. The patient was treated with atovaquone and azithromycin after she failed to respond to oral tetracycline and intravenous artemether treatment [21]. Although DNA amplification and microscopy were used for diagnosis, causality was not established. *Colpodella* spp. DNA was detected in a male patient with relapsing fever. The PCR-amplified DNA sequence was deposited in NCBI (accession number MF594625). However, characteristics of the infection were not reported. In a third human blood infection, a 28-year-old male ICU patient from Qiandongnan Prefecture, Guizhou Province, previously admitted into a tertiary hospital with recurrent fever of 1 week duration, was examined [22]. The patient had no recollection of a tick bite. Five days before admission, the patient had a fever, cough, and myalgia. Treatment with acetaminophen for three days did not clear the infection. Blood and sputum were collected for pathogen screening. *Colpodella* DNA was detected in the blood sample using next-generation sequencing (NGS). No hematological symptoms were observed. In the three human cases, there were no reports of bites from ticks or other arthropods (Table 2). Nymph stages of ticks actively transmit tick-borne pathogens. However, their very small size may be difficult to detect, and the human host may be unaware of the tick bite. The identified DNA from the ICU patient clustered with *Colpodella* spp. from Zambian cattle but did not cluster with DNA from pet dogs and *Rhipicephalus microplus* from Guizhou Province. Human adenovirus group B was detected in blood and sputum. Although the source of infection remained unknown, direct contact with infected companion animals exposes humans to pathogens. Huggins et al. [37] employed NGS in a Cambodian study to identify DNA sequences of bacteria and blood-borne pathogens from blood collected from 467 dogs. *Colpodella* spp. along with *P. caudatus* and *Bodo* spp. were identified in one dog. The DNA sequence had 95% sequence identity to *Colpodella* spp. identified in horse blood [23].

Four hundred horses from China were examined for blood-borne pathogens by PCR using primers targeting the 18S rRNA gene [23]. DNA from 2/400 horses had homology to DNA from *Colpodella* spp. related to *Colpodella* sp. ATCC 50594 and *Colpodella* strains HEP and HLJ. *Theileria* spp. (132/400) and *Babesia cabal* (2/400) were also identified in the horses. Huggins et al. [37] identified additional pathogens in arthropods (lice, fleas, and ticks) infesting dogs, including the apicomplexans *Babesia vogeli* and *Hepatozoon canis* and the kinetoplastids *Bodo* spp., *P. caudatus,* and *Trypanosoma evansi*. In addition to blood and CSF, *Colpodella gonderii* and *Colpoda steinii* were identified in urine from a female patient with a history of chronic diseases and urinary tract infection. Giemsa staining of the urine sample identified both protists. No other pathogens were identified, and no tick bites were reported. Treatment with ceftriaxone and metronidazole cleared the infection.

## 6. Colpodellosis with Gastrointestinal Symptoms

In colpodellosis reported in animals without recognized tick bites, direct infections through the drinking of contaminated water may not be the only route of infection. *Colpodella* spp. and *Colpoda* spp. were detected in fecal samples from sick and asymptomatic (healthy) Tibetan grazing ruminants in China. Seventy-nine fecal samples collected from free-range yak, Tibetan sheep, and a Tibetan goat were screened for the biodiversity of protists and nematodes in the animals by PCR [46]. Oligonucleotide primers targeting the V3-V4 fragment of the 18S rRNA gene were used for PCR, and NGS was performed to identify the DNA sequences. The dominant genera of parasites identified were *Entamoeba* (93.67%), *Blastocystis* (75.95%), *Trichostrongylus* (68.35%), *Colpoda* (50.63%), and *Colpodella* (49.37%). Sick animals had diarrhea with *Colpodella* spp. having a high prevalence in the sick animals investigated. Prevalence in asymptomatic animals for *Colpodella* spp. was 32.14%, and in sick animals was 92.86%. *Colpoda* spp. in asymptomatic animals was 39.29%, and in sick animals was 85.71%. *Colpodella* spp. prey on the ciliate *Colpoda* spp. The presence of both predator and prey in the stool samples indicates that both organisms are present in infected hosts. Both protists were identified in the urine of an infected human patient [29], and *Colpodella* spp. with the prey organisms *P. caudatus* and *Bodo* spp. were identified in the blood of an infected dog [37]. The presence of *Colpodella* spp. and its prey in fecal samples may reflect the release of the protists into the environment through fecal samples from infected hosts or the release of protists or infected ticks ingested during host feeding from the environment.

Alternatively, the protists may have contaminated fecal samples collected from the soil and may not be the cause of GI infections in the hosts. However, reports of *Colpodella* spp. DNA detection in fecal samples from ruminants, camels, foxes, and birds [47] is at least suggestive of opportunistic infections and needs to be investigated to determine whether *Colpodella* spp. like *Cryptosporidium* spp. cause GI symptoms. Molecular identification was used in the study by Wu et al. [46]. However, no morphological characterizations of *Colpodella* spp. were reported. Out of 19 total genera identified, the protists *Entamoeba* and *Colpodella* spp. were predominant.

*Cryptosporidium* spp. and *Colpodella* have been identified in coinfections using primers targeting the 18S rRNA gene of *Cryptosporidium* spp. It is important to unambiguously identify and distinguish oocysts of *Cryptosporidium* from cysts of *Colpodella* spp. in stool samples. *Colpodella* spp. cysts in the 4 nuclei stage may be misidentified as oocysts depending on the staining technique used. Polymerase chain reaction, NGS, and nucleic acid amplification techniques should be used alongside morphological identification methods for the identification of parasites. Life cycle stages initiating transmission, responsible for pathogenesis, and knowledge of sites of infection rely on morphological characterizations. Treatment options and success depend on knowledge of life cycle stages. Treatment effective for the trophozoite stage may be ineffective for cysts. Tick bites were not reported in this study, and the reported symptoms are for GI tract infection. Diarrhea and fever were among the symptoms in sick two-humped camels infected by *Colpodella* spp. through tick bites [25]. *Colpodella* spp. was identified in fecal samples collected from red fox (*Vulpes vulpes*) [15] at the Hebei Xiaowutai Mountain National Nature Reserve. The suspected source of transmission was drinking water [15]. Ticks are thought to pick up *Colpodella* spp. from water and then transmit the parasite to human and animal hosts [11]. Hasapis et al. [47] identified *Colpodella* spp. in fecal samples of birds, ruminants, and a fox phylogenetically related to *Colpodella* strain HEP, HLJ, and strains from *Colpodella* identified in sheep from Nigeria [20]. The Nigerian goats and sheep were screened by PCR for *Cryptosporidium* spp. using primers targeting *Cryptosporidium* spp. and *Colpodella* spp. DNA was identified. The DNA identified had sequence identity with the 18S rRNA gene of *Colpodella* spp. from Cyprus, found in a duck and a fox. *Cryptosporidium* spp. oocysts were identified by microscopic examination. The ruminants had diarrhea [18]. Similarly, fecal samples collected from asymptomatic large cats from the Harbin Zoo, China screened for *Cryptosporidium* spp. using 18S rRNA identified *Colpodella* spp. [19]. No tick bites were reported.

## 7. Tick Vector Competence for Pathogen Transmission

Although time-consuming in some cases and requiring expert microscopists for accurate identification of morphological features of parasites, distinguishing the morphology of the species and strains of *Colpodella* spp. identified by DNA is crucial in furthering our understanding of parasite life cycle stages present in infections. Many tick-borne infections present with similar symptoms early in infection [30]. Accurate diagnosis of infection in the case of multiple tickborne pathogens present in the host at the same time will require a knowledge of incubation times, characteristic pathognomonic signs, if known, and accurate identification of pathogen morphology to aid molecular diagnosis [30,48,49]. Diagnostic methods comprising staining, microscopy, and molecular techniques have been used for the diagnosis of known *Cryptosporidium* species and used to identify novel species [50]. Tick-borne infections present with gastrointestinal disturbances and should be considered in cases where *Colpodella* spp. are identified with an unknown source of transmission [51]. Co-infections of tick-borne pathogens present challenges in recognizing specific symptoms. However, if symptoms persist following treatment for a suspected tickborne pathogen, coinfection with other pathogens should be suspected. A case of a 70-year-old man diagnosed with Lyme disease, anaplasmosis, and babesiosis, illustrates the pattern of coinfection that can occur in human and animal hosts with tickborne pathogens [49]. Colpodellosis as the result of coinfections and single infections has been reported [10,11,12,13,14,15,17,19,20,21,22,23,24,25,26]. Phylogenetic analysis of *Colpodella* spp. DNA demonstrates distinct clades of *Colpodella* spp. reflecting different species, strains, and patterns of virulence [52].

In symptomatic animal and human infections where *Colpodella* spp. DNA was amplified from blood and CSF, respectively, the contributions of *Colpodella* spp. life cycle stages to pathogenesis are unknown. What features of the tick enhance vector competence for the possible transmission of *Colpodella* spp. life cycle stages to vertebrate hosts? Studies reporting tick vector incompetence show that even tick species known to be competent biological vectors for some species of *Babesia* are not competent for other species [53]. The development of the tick midgut during metamorphosis, known as hemimetabolus metamorphosis, provides an environment supportive of parasite development through the molting phase of the tick [54]. Parasites infecting the tick can survive through the life cycle stages of the tick if they infect the ovaries in transovarial transmission. The parasites can survive and be transmitted from the eggs to larvae, nymphs, and adult stages in transstadial vertical transmission. Life cycle stages are hematophagous, requiring a blood meal at each life cycle stage, where the ticks can pick up pathogens present in the blood of a host, and can transmit the pathogen to a new host during feeding in trans-stadial horizontal transmission [54]. Among the ticks reported to carry *Colpodella* spp., the Ixodidae (hard-bodied ticks) family has been most investigated to understand vector capacity and vector competence [54]. Ixodid ticks are multi-host ticks that feed on multiple vertebrate hosts throughout their life cycles [54,55]. They are found in diverse habitats across different countries; they feed frequently and ingest large blood meals followed by long digestion periods of weeks to months [54,55]. Pathogens picked up by the tick can move from the midgut to various tissues and organs of the tick, including entry and invasion of the salivary glands, and presence in the tick saliva, where trans-stadial horizontal transmission can occur to vertebrate hosts. Life cycle stages of pathogens, such as gametes, oocysts, trophozoites, or cysts, can alter tick physiology, leading to changes in tick metabolic activity, mobility, survival, reproduction, and molting as well as changes to the microbiome within ticks [54,55]. Such changes can enhance pathogen proliferation and survival as well as influence tick vector competence [54,55]. The sexual cycle of pathogens like *Babesia* spp. and *Theileria* spp. occur within the midgut of the biological tick vector host with transfer of sporozoite stages to the salivary glands, where they become infective [27]. Host-seeking behavior known as “questing,” in which all motile stages, such as nymphs, climb to the tops of vegetation and latch onto vertebrate hosts that brush against it, allows the tick to find a new blood source while transmitting pathogens during feeding.

It is crucial to investigate tick vector competence for *Colpodella* spp. transmission to know which of the tick genera reported to carry *Colpodella* spp. represent competent vectors with the capacity to spread *Colpodella* spp. among human and animal hosts. Currently, vector competence for *Colpodella* spp. transmission is unknown. Genera of the Ixodidae family that have been reported to carry *Colpodella* spp. include *Ixodes*, *Haemaphysalis*, *Amblyomma*, *Rhipicephalus*, *Hyalomma,* and *Dermacentor* (Table 1). *Ixodes scapularis* transmits *B. burgdorferi* for Lyme disease and *B. microti* for babesiosis. *Ixodes scapularis*, *I. ricinus,* and *I. cookei* transmit Powassan virus (POWV), which causes encephalitis and meningitis [55]. *Amblyomma americanum* transmits *Ehrlichia chaffeensis,* which causes ehrlichiosis, and *Dermacentor andersoni,* along with *Rhipicephalus sanguineus,* transmit *Rickettsia rickettsii* for Rocky Mountain Spotted Fever. *Colpodella* spp. DNA has been amplified from the same ticks containing *Babesia* spp., *Ehrlichia* spp., and *Rickettsia* spp. [10,25,43]. Even in well-established cases of tick-borne pathogen transmission, particularly in coinfections, the dynamics of coinfection within the tick and in humans are highly complex, with factors from the vertebrate host immune system influencing host susceptibility and resistance to infection [56]. The interactions of coinfecting pathogens within the tick can impact tick fitness, alter the tick microbiome, modulate innate immune responses in the tick, resulting in immune evasion and epigenetic regulation, which results in the inhibition of apoptosis, thereby facilitating infection by pathogens [55,57]. In the vertebrate host, coinfections can modulate pathogenesis, tissue destruction, and disease severity [56,57].

Amplification of *Colpodella* spp. DNA from ticks and specimens from vertebrate hosts is not enough to establish *Colpodella* spp. as the infective pathogen if there is no demonstration of viable life cycle stages of *Colpodella* spp. in the tick tissues, such as in hemolymph from adult ticks, salivary glands, ovaries, or larval stages of the tick. If DNA is amplified from ticks but not from the vertebrate host harboring the ticks, might the DNA amplification be from dead *Colpodella* spp. in the tick? Much remains unknown about *Colpodella* spp. biology. The descriptions of potential transmission routes for tick transmission of *Colpodella* spp. proposed in this review (Figure 1), while speculative, provides a guide for investigating *Colpodella* spp. transmission mechanisms. *Colpodella* spp. DNA has been amplified using PCR primers targeting the 18S rRNA gene of *Babesia* spp. and *Theileria* spp. Non-specific targeting and cross-amplification can occur with the use of these primers. Although the transmission mechanism of *Colpodella* spp. is currently unknown, the apicomplexan similarities present an opportunity to determine if the transmission of *Colpodella* spp. to animals and human hosts is like that of the piroplasms. Specific *Colpodella* spp. molecular markers need to be identified to aid in the identification of *Colpodella* spp. The transmission mechanisms for *Colpodella* spp. in ticks may be very different from what we know for apicomplexan parasites like the piroplasms transmitted by ticks. Virulence molecules are unknown for *Colpodella* spp. Tick bites in and of themselves are not evidence for infection, but rather the amplification of *Colpodella* spp. DNA and the presence of viable *Colpodella* stages shows evidence of infection. Identifying viable *Colpodella* spp. life cycle stages using staining and microscopy, performing in vivo lab studies in animal models and in ticks to determine infection and pathogenesis, and culturing *Colpodella* spp. from specimens collected from ticks and vertebrate hosts are of paramount importance. Examination of tick eggs and larval stages for *Colpodella* cysts and trophozoites, including examinations of the midgut and salivary glands for viable *Colpodella* spp. will provide unambiguous identification of *Colpodella* spp. in the tick and in the vertebrate hosts.

An integrated approach for diagnosis will allow the identification of *Colpodella* spp. in single infections and the identification of *Colpodella* spp. with multiple coinfecting pathogens at the same time [30]. The use of multiplex assays and molecular techniques like fluorescent in situ hybridization (FISH) techniques [58] may further our ability to enhance morphological observations of life cycle stages, along with staining protocols. The extent of *Colpodella* spp. infections in diverse animals, their transmission by ticks [10,11,12,14,17,25,45,59], and the diverse tissue locations where *Colpodella* spp. DNA has been identified [21,22,23,24,60], underscores the urgency of intensified efforts to improve our understanding of this emerging tickborne pathogen and important public health risk.

## 8. Conclusions and Recommendations

Increased reports of colpodellosis in humans and animals have provided further insights into the disease’s symptoms. However, the presence of multiple pathogens within ticks and the lack of knowledge regarding the life cycle stages of *Colpodella* spp. mediating transmission, spread, and pathogenesis in hosts is a major obstacle in furthering our understanding of this disease. The increased reports of *Colpodella* spp. detection in ticks may be due to more epidemiological screenings being conducted to survey piroplasm abundance among wildlife, agricultural, and domestic animals. Alternatively, the increase in detection may reflect increased prevalence of *Colpodella* spp. due to environmental factors influenced by climate change. Detection of *Colpodella* spp. DNA has been useful in identifying *Colpodella* spp. as an infective organism, particularly in the vertebrate host infections where only *Colpodella* spp. was identified. However, without morphological characterization and serological evaluation to aid molecular diagnosis, key aspects of disease development and pathogenesis remain unknown. Staining tick hemolymph and other tissues, host blood, and fecal samples to identify *Colpodella* spp. along with coinfecting protist pathogens is vital. The presence of prey protists for *Colpodella* spp. can also be identified. *Colpodella* spp. should be suspected in cases of babesia-like illnesses unresponsive to conventional treatment. In cases of a single *Colpodella* spp. infections, the presence of protist prey such as ciliates, bodonids, and algae should be investigated using primers targeting the 18S rRNA genes of the prey. In cases of diarrhea suspected to be caused by *Cryptosporidium* spp., *Colpodella* spp. should also be considered. Fecal samples should be examined for both oocysts and cysts. Samples containing *Colpodella* spp. DNA should be tested in experimental studies to determine if lab animal models can become infected by *Colpodella* spp. and whether uninfected ticks can acquire infections from the host. Studies to establish vector competence are needed to confirm tick vector capacity. Identification of molecular markers for transmission and virulence will aid in understanding the biology of *Colpodella* spp. Integration of diagnostic methods that enhance point-of-care diagnostics will create new opportunities to robustly apply advanced morphological and molecular techniques to understand the biology of *Colpodella* spp. This will benefit treatment and prevention and reduce the risk of *Colpodella* spp. human infections.

## Figures and Tables

**Table 1:** Detection of *Colpodella* spp. DNA in ticks infesting humans and animals from different geographic locations.

Ticks	Geographic Location	Detection Method	References
Human infection			
*Ixodes persulcatus*	Heilongjiang Province, Northeast China	PCR	[14]
Cattle			
*Rhipicephalus (Boophilus) microplus*	Private farms in the Nacala Porto and Monapo districts of Nampula province, Mozambique	PCR	NCBI access number KY914473
*Rh. bursa*	Free grazing cattle in the Puglia region in Southern Italy	PCR	[45]
Malayan pangolins			
*Amblyomma javanense*	Guandong Provincial Wildlife Rescue Center at Guangzhou Zoo and Guandong Institute of Applied Biological Resources, China	PCR	[10]
South China Tiger			
*Rh. duttoni*	The Meihua Mountains, Fujian China	PCR	[11]
*Haemaphysalis longicornis*			
*H. flava*			
*H. bispinosa*			
*H. hystricis*			
*D. andersoni*			
*D. atrosignatus*			
*D. taiwanensis*			
Dromedary Camels			
*Hyalomma dromedarii*	Aswan and Luxor Governorates, Southern Egypt	PCR	[10]
Cattle			
*Rh. annulatus*	Qena, Sohag and Luxor Governorates, Southern Egypt	PCR	[44]
Goats			
*Haemaphysalis longicornis*	Yiyuan County, Shadong province, China	PCR	[17]
Goats			
*Hy. anatolicum*	Districts of Khyber Pakhtunkhwa, Pakistan	PCR	[43]
*Hy. excavatum*			
Two-humped Camels			[25]
*Haemaphysalis longicornis*	Gaotai County, Gansu Province, China	PCR	
*Hy. asiaticum*			
Ticks			
*Rh. haemaphysalides*	Qinghai Province, northwest China	PCR	NCBI Access. number MH208621
*Dermacentor everestianus*			NCBI access. number MH012047
*D. nuttalli*			NCBI access. number MH012045
Tick	Yunnan, China	Genomic DNA	
*Ixodes acutitarsus*			NCBI access. Number PX220049
*Ixodes sinensis*			NCBI access. number PX220047
*Ixodes granulatus*			NCBI access. Number PX220017
*Ixodes ovatus*			NCBI access. Number PX220014
*Amblyomma testudinarium*			NCBI access. Number PX220048
*Haemaphysalis montgomeryi*			NCBI access. Number PX220044
*Haemaphysalis nepalensis*			NCBI access. number PX220018

**Table 2:** Symptomatic and asymptomatic *Colpodella* spp. infections reported in humans and animals.

*Colpodella* spp. in Humans and Animals	Year	Country	Reference
Tickborne *Colpodella* spp. infections			
Human infection			
Human tickborne infection, neurological symptoms, single infection, female, fever, dizziness, gait disturbance, headache	2018	China	[14]
Animal infections			
Tiger (*Panthera tigris amoyensis* Hizheimer) in blood and ticks, tickborne *Colpodella* spp. single infection, anorexia, runny nose, drool, bluish-green stool, at autopsy multiple organ damage	2022	China	[11]
Two-humped camels (*Camelus bacterianus*), *Colpodella* spp. in blood and infesting ticks, fever, appetite loss, diarrhea, fatigue, decreased milk output	2025	China	[25]
Pangolins, *Colpodella* spp. in infesting ticks, co-infection, anorexia, cough, edema of extremities, drowsiness, at autopsy severe organ damage, congestion, edema of major organs, ascites, inflammation	2024	China	[10]
Camels, *Colpodella* spp. in infesting ticks, asymptomatic infection	2024	Egypt	[12]
Cattle and goats, *Colpodella* spp. in infesting ticks, asymptomatic infection	2024	Italy	[45]
Goats and dogs, *Colpodella* spp. in ticks, asymptomatic infection	2024	China	[17]
Cattle, tick associated *Colpodella* spp. infection, co-infection	2017	Mozambique	NCBI access number KY914473
Goats, *Colpodella* spp. in infesting ticks	2026	Pakistan	[43]
*Colpodella* spp. in a biting fly			
Horse, *Colpopdella* spp. in infesting biting fly (*Stomoxys indicus*), co-infection	2023	Thailand	[13]
*Colpodella* spp. in blood infections			
Human infections			
Human relapsing fever, non-tick associated blood infection, single *Colpodella* spp. infection, female, productive cough, malaise, hemolytic anemia	2012	China	[21]
Human relapsing fever, non-tick associated, single *Colpodella* spp. infection, male	2017	China	NCBI accession number MF594625
Human relapsing fever, non-tick associated *Colpodella* spp. blood infection, male, fever, cough, myalgia	2025	China	[22]
Animal infections			
Cat, non-tick associated *Colpodella* spp. single blood infection, inflammation, tissue damage	2023	USA	[24]
Cattle and wildlife, non-tick associated *Colpodella* spp. blood infection, co-infection, asymptomatic	2020	Zambia	[18]
Horse non-tick associated *Colpodella* spp. blood infection, co-infection, asymptomatic	2022	China	[23]
Dog, non-tick associated *Colpodella* spp. blood infection, co-infection, *Parabodo caudatus* and *Bodo* spp. prey for *Colpodella* spp., asymptomatic	2021	Cambodia	[37]
Cats and dogs, non-tick associated *Colpodella* spp. blood infection, co-infection, asymptomatic	2023	China	[26]
*Colpodella* spp. in GI tract infections			
Animal infections			
Goats and sheep, non-tick associated infection, *Colpodella* spp. in diarrhetic fecal samples, co-infection	2024	Nigeria	[20]
Tibetan sheep, goat and yak, *Colpodella* spp. and prey *Colpoda* spp. in fecal samples from asymptomatic and diarrhetic animals	2025	China	[46]
Goats, fox, duck, Eurasian Coot, non-tick associated, *Colpodella* spp. in fecal samples	2025	Cyprus	[47]
Large zoo felids, *Colpodella* spp. in fecal samples, co-infection, asymptomatic	2021	China	[19]
*Colpodella* spp. in urinary tract infection			
Human infection			
Human urinary tract infection associated with *Colpodella gonderi* and its prey *Colpoda steinii*, female	2021	Romania	[29]
*Colpodella* spp. infection in skin			
Animal infection			
Raccoon, non-tick associated *Colpodella* spp. in the skin of the ear, co-infection	2019	Poland	NCBI accession number MN103991

## Data Availability

No new data were created or analyzed in this study.
